# Intra-articular Steroid Injection as the Etiology of Acute Pancreatitis

**DOI:** 10.7759/cureus.59995

**Published:** 2024-05-09

**Authors:** Sareena Ali, Richard Galka, Alan Shapiro

**Affiliations:** 1 Internal Medicine, Advocate Lutheran General Hospital, Park Ridge, USA; 2 Imaging Services, Advocate Lutheran General Hospital, Park Ridge, USA; 3 Gastroenterology, Advocate Lutheran General Hospital, Park Ridge, USA

**Keywords:** acute pancreatitis (ap), adult gastroenterology, steroid injection, drug-induced acute pancreatitis, pancreatitis

## Abstract

Acute interstitial pancreatitis is typically caused by gallstones and alcohol use. Less common causes include infection and drugs. Patients present with epigastric pain and often require pain medications and hospitalization depending on severity. We present a unique case of drug-induced pancreatitis likely caused by intra-articular corticosteroid injections on two separate occasions in the same patient. In both instances, other etiologies were ruled out. Given the temporal relationship between the intra-articular corticosteroid injection and presentation of pancreatitis, the corticosteroid injection was the likely etiology. This case suggests that intra-articular steroids should be included as an etiology of drug-induced pancreatitis.

## Introduction

In the United States, acute pancreatitis continues to be one of the leading gastrointestinal causes of hospitalization. Acute interstitial pancreatitis is characterized by inflammation of the pancreatic parenchyma and peripancreatic tissues. Patients present with epigastric pain that ranges from mild to severe in intensity. Etiologies most commonly include gallstones and alcohol use, while less common causes are infection, autoimmune, and drug-induced [1]. Medications are the cause of acute pancreatitis in about 0.1-2% of cases. Oral corticosteroids are a known cause of drug-induced pancreatitis. Previous research indicates that oral steroids can cause alteration of lipid and calcium metabolism or increase the viscosity of pancreatic secretions and delay emptying, leading to acute pancreatitis [2-3]. Intra-articular steroids have been implicated as a cause of pancreatitis only once in a Letter to the Editor [4]. Intra-articular steroids may be systemically absorbed and have been noted to affect the hypothalamus-pituitary-adrenal axis, but this is not known to be an etiology of pancreatitis [5]. We present a unique case of two instances of drug-induced pancreatitis in the same patient, likely due to intra-articular corticosteroid injection use from betamethasone. This case was presented as a poster presentation at the ACG conference on October 22nd, 2023 in Vancouver, Canada.

## Case presentation

**Figure 1:**
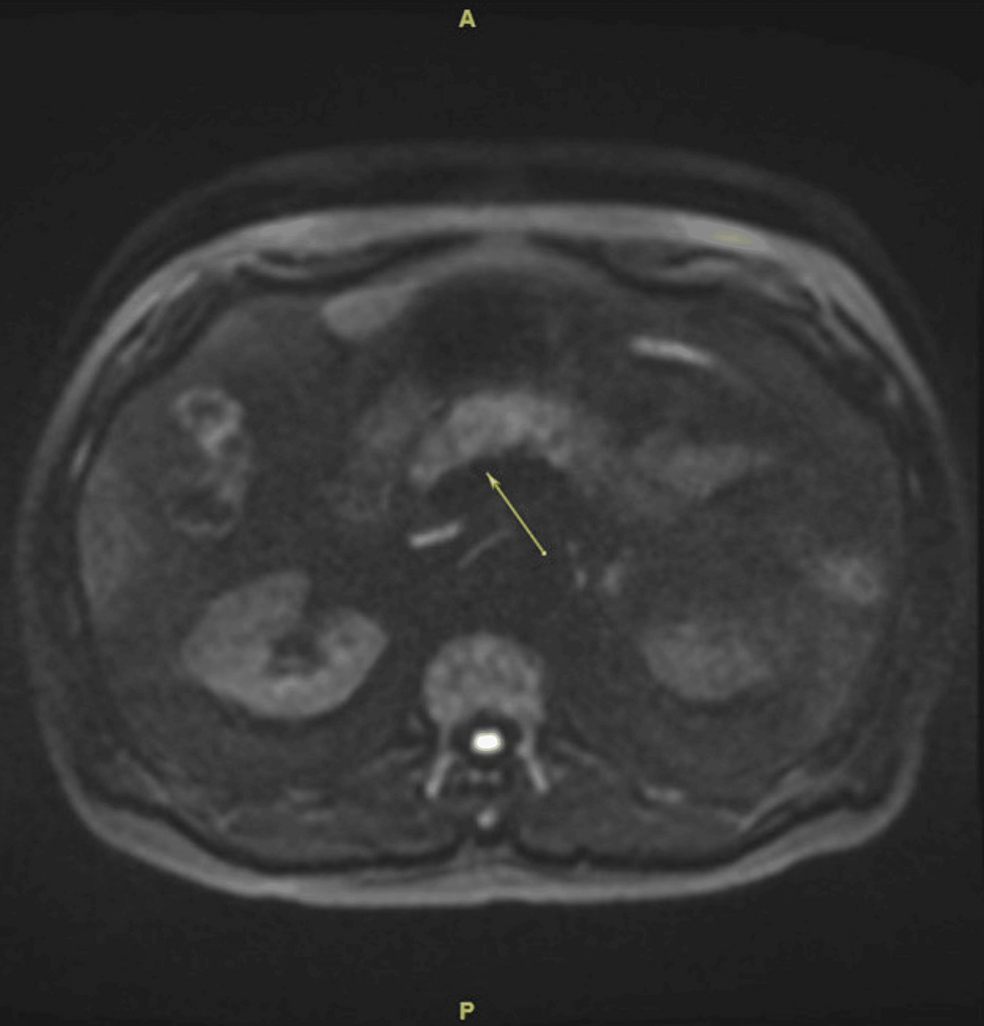
MRCP image from the first episode of pancreatitis From the first episode of pancreatitis, Figure 1 is an MRCP image that shows a mildly increased DWI signal within the pancreatic head and body (arrow). This is radiographic evidence of acute pancreatitis given the patient's clinical presentation. MRCP: Magnetic resonance cholangiopancreatography; DWI: Diffusion-weighted imaging

**Figure 2:**
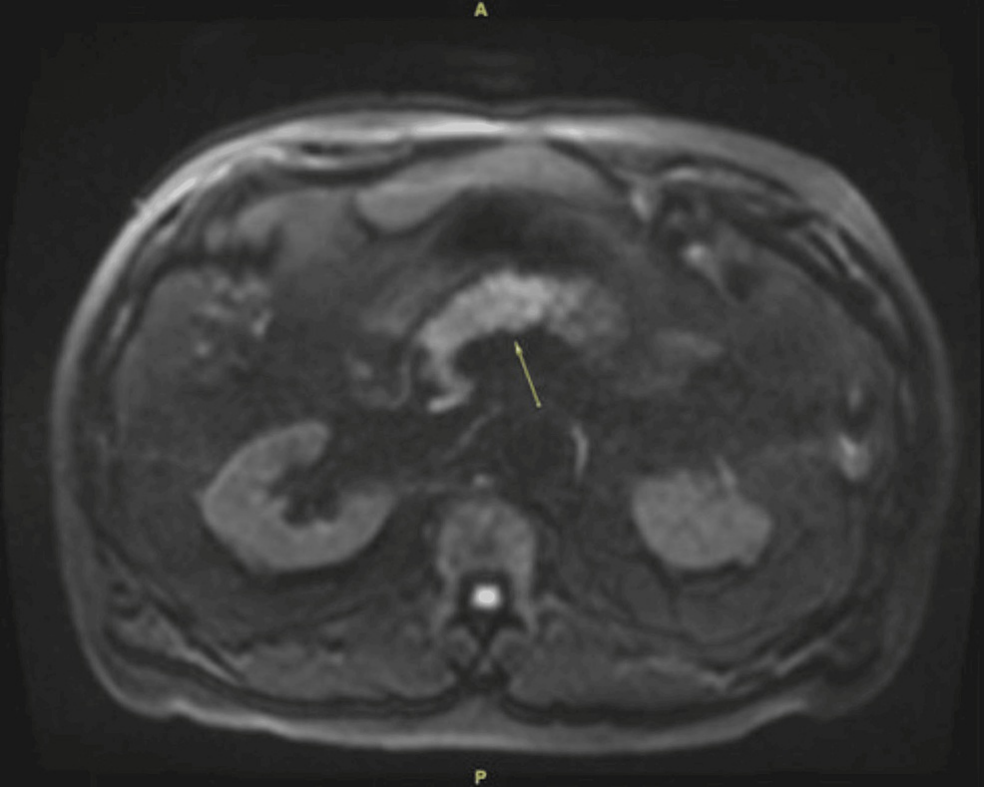
MRCP image from the second episode of pancreatitis Figure 2 shows an MRCP image from the second episode of pancreatitis. There is again a mildly increased DWI signal in the pancreatic head and body in Figure 2 (arrow), which signifies acute pancreatitis. MRCP: Magnetic resonance cholangiopancreatography; DWI: Diffusion-weighted imaging

A 53-year-old male with a past medical history of eosinophilic esophagitis (EoE) presented with a one-week history of epigastric pain. Three days prior to onset, the patient had an intra-articular betamethasone injection to his right shoulder due to biceps tendonitis. He had been on chronic swallowed fluticasone 440 mcg daily and budesonide 1 mg daily for EoE, but there were no new oral medications at this time. He recalled receiving a 2cc triamcinolone injection in the cervical spine previously without any development of these symptoms. Upon presentation with epigastric pain, physical examination at that time showed no distention, normal bowel sounds, and mild epigastric tenderness without rebound. Amylase and lipase were elevated to 136 U/L and 1668 U/L, respectively. C-reactive protein (CRP) was initially elevated to 31.41 mg/L. Liver function tests were within normal limits. The patient had a magnetic resonance cholangiopancreatography (MRCP) which showed findings consistent with mild acute interstitial pancreatitis, a small 7 mm bilobed pancreatic cyst, no pancreatic ductal dilatation, no biliary ductal dilatation, and no choledocholithiasis (Figure 1). Etiology was unknown as he did not have any history of alcohol use, had a prior cholecystectomy, and no known offending medications. IgG4 level was checked and was normal at 48 mg/dL, and triglycerides were 130 mg/dL. Upper endoscopy with endoscopic ultrasound (EUS) was performed due to unexplained pancreatitis and concern for a cyst. Esophagogastroduodenoscopy (EGD) showed a few subtle rings in the esophagus with a normal stomach and duodenum. EUS showed normal parenchyma of the pancreas without any visualized mass lesion or cyst and a non-dilated main pancreatic duct. The common bile duct was nondilated (4.3 mm) and was normal, with no stones or shadows noted. This episode of pancreatitis resolved within one week, without hospitalization or need for narcotics. The etiology was still unclear, other than the patient having an intra-articular corticosteroid injection prior to pancreatitis. Two years later, he again presented with epigastric pain and nausea three days after receiving a 6mg betamethasone injection in his left shoulder. The patient did not have any intra-articular steroid injections in between this time frame. Lipase was greater than 5000 U/L and MRCP at this time showed mild interstitial pancreatitis with normal biliary ducts, no evidence of pancreas divisum, and stable cystic foci in the pancreas (Figure 2). The epigastric pain lasted for three days, and he did not require hospitalization.

## Discussion

We present a unique case of intra-articular corticosteroid injection as the probable cause of pancreatitis. Systemic steroids are known to be a cause of drug-induced pancreatitis and the risk of development is usually highest four to 14 days after taking this medication. The pathophysiology of how steroids cause pancreatitis is still poorly understood, but current research indicates that steroids increase the viscosity of pancreatic secretions and delay emptying [3, 6]. Intra-articular steroid injections as a cause has only been reported once before in a Letter to the Editor [4]. There is one report of a case of pancreatitis due to a lumbar epidural steroid injection [7]. Our patient had been on long-term topical fluticasone and budesonide for EoE without any episodes of pancreatitis, so this is not likely the etiology. It is interesting that our patient received a triamcinolone injection in the past, which has been shown to have more of a systemic effect, but did not develop pancreatitis. However, the effect of betamethasone versus triamcinolone could have occurred due to differences in dosage, type of steroid, and systemic absorption between these two agents [8-10]. There have been previous case reports of systemic effects of intra-articular corticosteroids, but these typically resulted in disorders such as transient suppression of the hypothalamus-pituitary-adrenal axis [5, 9].

Our patient developed acute pancreatitis after receiving an intra-articular betamethasone injection. A tool that can help with estimating the likelihood of an adverse drug reaction is the Naranjo score. The score range is from -4 to 13. If the score is 9 or higher, there is a definite reaction, scores of 5-8 are probable and scores of 1-4 are possible. It is less likely the drug caused a reaction if the score is 0 or less. For our patient, the Naranjo score is 10 (definite) (Table 1) [11]. Lending support to causality is that our patient was inadvertently rechallenged with a second intra-articular steroid injection, resulting in another episode of pancreatitis. In both instances, other etiologies of acute pancreatitis were ruled out, making the betamethasone injection the suspected cause. He did not receive any new oral medications that could have caused him to develop acute pancreatitis. Additionally, both instances were not severe, as they did not require hospitalization. As described previously, there is only one prior report in the form of a Letter to the Editor describing intra-articular corticosteroid injection as the cause of acute pancreatitis [4]. Given the temporal relationship between intra-articular steroid injection and the development of pancreatitis on two separate occasions, betamethasone injection is the likely etiology.

**Table 1:** Naranjo Scale This includes the Naranjo scale that is filled out, showing acute pancreatitis as an adverse drug reaction of intra-articular betamethasone injection.

Naranjo Adverse Drug Reaction Probability Scale				
Question	Yes	No	Do Not Know	Score
1. Are there previous conclusive reports on this reaction?	1	0	0	1
2. Did the adverse event appear after the suspected drug was administered?	2	-1	0	2
3. Did the adverse reaction improve when the drug was discontinued or a specific antagonist was administered?	1	0	0	1
4. Did the adverse event reappear when the drug was re‐administered?	2	-1	0	2
5. Are there alternative causes (other than the drug) that could on their own have caused the reaction?	-1	2	0	2
6. Did the reaction reappear when a placebo was given?	-1	1	0	0
7. Was the drug detected in blood (or other fluids) in concentrations known to be toxic?	1	0	0	0
8. Was the reaction more severe when the dose was increased or less severe when the dose was decreased?	1	0	0	0
9. Did the patient have a similar reaction to the same or similar drugs in any previous exposure?	1	0	0	1
10. Was the adverse event confirmed by any objective evidence?	1	0	0	1
Total Score				10

## Conclusions

Here, we report only the second well-documented case of intra-articular corticosteroid injection as a cause of acute pancreatitis. Therefore, perhaps intra-articular steroids should be added to the list of etiologies of drug-induced pancreatitis. Pancreatitis should also be considered as a possible adverse effect when obtaining informed consent for local steroid injections. Clinicians should have a high index of suspicion if pancreatitis does develop after an intra-articular injection of steroids, so that future episodes are avoided.
